# Enhancing CO_2_ adsorption capacity of ZIF-8 by synergetic effect of high pressure and temperature

**DOI:** 10.1038/s41598-023-44960-4

**Published:** 2023-10-16

**Authors:** Shan Jiang, Jingyan Liu, Jiwen Guan, Xin Du, Shoushun Chen, Yang Song, Yining Huang

**Affiliations:** 1 Department of Chemistry, The University of Western Ontario, London, ON N6A 5B7 Canada; 2 Department of Physics and Astronomy, The University of Western Ontario, London, ON N6A 5B7 Canada; 3 College of Chemistry and Chemical Engineering, Lanzhou Magnetic Resonance Center, Lanzhou University, Lanzhou, 730000 China

**Keywords:** Condensed-matter physics, Materials for energy and catalysis, Soft materials, Physical chemistry, Chemical physics, Chemical physics, Condensed-matter physics

## Abstract

Metal–organic frameworks (MOFs) and zeolitic imidazolate frameworks (ZIFs) are promising porous materials for adsorption and storage of greenhouse gases, especially CO_2_. In this study, guided by the CO_2_ phase diagram, we explore the adsorption behavior of solid CO_2_ loaded with ZIF-8 framework by heating the sample under high pressures, resulting in a drastic improvement in the CO_2_ uptake. The behavior of CO_2_ under simultaneous high temperature (T) and pressure (P) conditions is directly monitored by in situ FTIR spectroscopy. The remarkable enhancement in CO_2_ adsorption capability observed can be attributed to the synergetic effect of high T and P: high temperature greatly enhances the transport property of solid CO_2_ by facilitating its diffusion into the framework; high pressure effectively modifies the pore size and shape via changing the linker orientation and creating new adsorption sites within ZIF-8. Our study thus provides important new insights into the tunability and enhancement of CO_2_ adsorptive capability in MOFs/ZIFs using pressure and temperature combined as a synergetic approach.

## Introduction

To address the challenges associated with global warming, greenhouse gases and especially carbon dioxide capture and storage is of fundament importance. Compared to the low energy efficient chemisorption-based methods, solid physisorbent materials such as activated carbons, zeolites, metal–organic frameworks (MOFs) and zeolitic imidazolate frameworks (ZIFs), which have lower heat capacities and require lower regeneration energy, have attracted increasing attention. In particular, MOFs and ZIFs as a subclass of MOFs have emerged as promising porous materials for greenhouse gas capture and storage. These materials have unique properties such as very high surface area, well defined porosity, high chemical and structural stability along with their modularity and tunable pore size/functionality^1,2^. All these properties present remarkable potential for achieving optimal CO_2_ capture and storage performance. In the large ZIF family, ZIF-8 [Zn(MeIm)_2_, MeIm = 2-methylimidazolate] is the most well-known member. ZIF-8 is constructed by connecting each zinc ion tetrahedrally to four individual methylimidazolate ligands. It has a sodalite (SOD) topology containing the cages with a diameter of 11.6 Å and a cage aperture of 3.4 Å (Supplementary Fig. S1)^3^. These specific parameters make ZIF-8 to exhibit excellent adsorptive ability towards small gas molecules with appropriate kinetic diameters^4^, whose structural properties and gas adsorption performance including CO_2_ have been extensively studied in the past decade under ambient conditions, high external pressures and at low temperatures by experimental and computational methods^5–8^.

Recent investigations of pressure effect in different classes of MOFs are of particular interest which revealed highly diversified structural behaviours^9–20^. Among these, ZIFs and especially ZIF-8 under high external pressure have been examined extensively^21–28^. More significantly, several studies have shown that application of high external pressure can effectively tune CO_2_ storage capability in MOFs/ZIFs^8,29–32^. This is because external pressure can change the MOF/ZIFs framework topology, alter pore size and shape, enhance host–guest interactions between framework and adsorbed CO_2_, and even create new adsorption sites, leading to an increased CO_2_ adsorptive capability. However, at room temperature, solidification of CO_2_ occurs at a pressure above 0.6 GPa^33^. This phase change of CO_2_ severely limits further insertion of CO_2_ into the cavities of ZIF-8 at higher pressures since solid CO_2_ is immobile and not diffusible. This problem can be remediated if a mixture of solid CO_2_ and ZIF-8 is heated to a temperature at which solid CO_2_ existing outside ZIF-8 is mobilized, so that a significantly larger number of CO_2_ molecules may be pressed into the framework of ZIFs by pressure. More importantly, the detailed structural information, pressure and temperature stability, as well as the CO_2_ adsorption properties in ZIF-8 established in previous studies allows the understanding of the possible synergetic high-P and high-T effect on CO_2_ adsorption in ZIF-8.In this work, using CO_2_ phase diagram^33^ as a guide and in situ IR spectroscopy as a tool, we examined CO_2_ adsorption behaviour of ZIF-8 in a diamond-anvil cell (DAC) by simultaneously applying high temperature and pressure. To the best of our knowledge, this is the first report that investigates the CO_2_ intake behaviour at simultaneous high-P and high-T conditions for MOFs and especially for ZIF-8. Our results clearly show that under the carefully controlled high P–T conditions, CO_2_ uptake of ZIF-8 is enhanced drastically, making it a promising material for CO_2_ storage.

## Results

**Figure 1:**
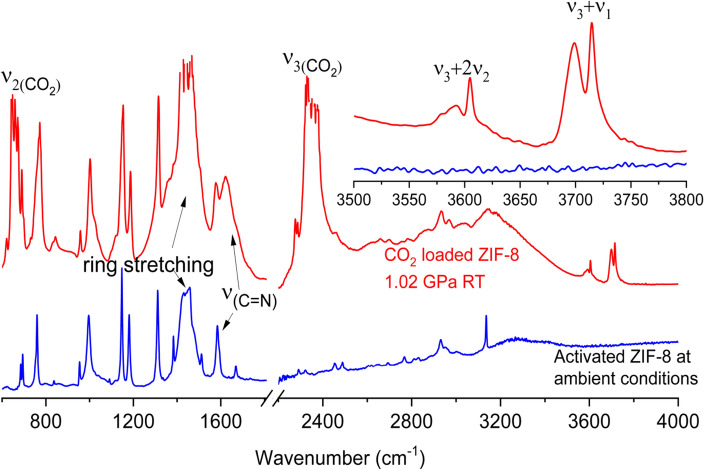
Mid-IR spectra of activated ZIF-8 at ambient pressure (blue) and CO_2_ loaded ZIF-8 at 1.02 GPa (red). The inset shows the comparison between the activated (bottom) and CO_2_ loaded ZIF-8 (top) in the spectral region of the CO_2_ combination modes.

A typical IR spectrum of a mixture of CO_2_ and activated (empty) ZIF-8 loaded into a DAC (hereafter referred to as CO_2_/ZIF-8) under pressure (e.g., 1.02 GPa) along with the spectrum of activated ZIF-8 is shown in Fig. 1. The observation of very strong CO_2_ fundamental modes, ν_2_ and ν_3_, confirms that CO_2_ was successfully loaded into the DAC. However, these peaks are too intense and saturated, preventing further data analysis. As shown in literature work^34,35^, in such a situation, two CO_2_ combination modes, (ν_3_ + 2ν_2_) and (ν_3_ + ν_1_) observed at around 3600 and 3710 cm^-1^ in the IR spectra can be more effectively used to monitor the behaviour of adsorbed CO_2_ inside porous framework^29,30^. It is worth noting that at 1.02 GPa, each combination mode split into a doublet. The shape and position of the sharper high-frequency component for each mode are almost identical to those of pure CO_2_, suggesting that it is due to the CO_2_ outside the ZIF-8 framework in solid phase, whereas the boarder low-frequency component is due to the CO_2_ molecules that have entered the framework of ZIF-8 similar to that observed in other MOFs we reported previously^29,30^. The relative intensity of these bands forms the basis for quantitative analysis of CO_2_ in different environment under specific pressure and temperature conditions.

**Figure 2:**
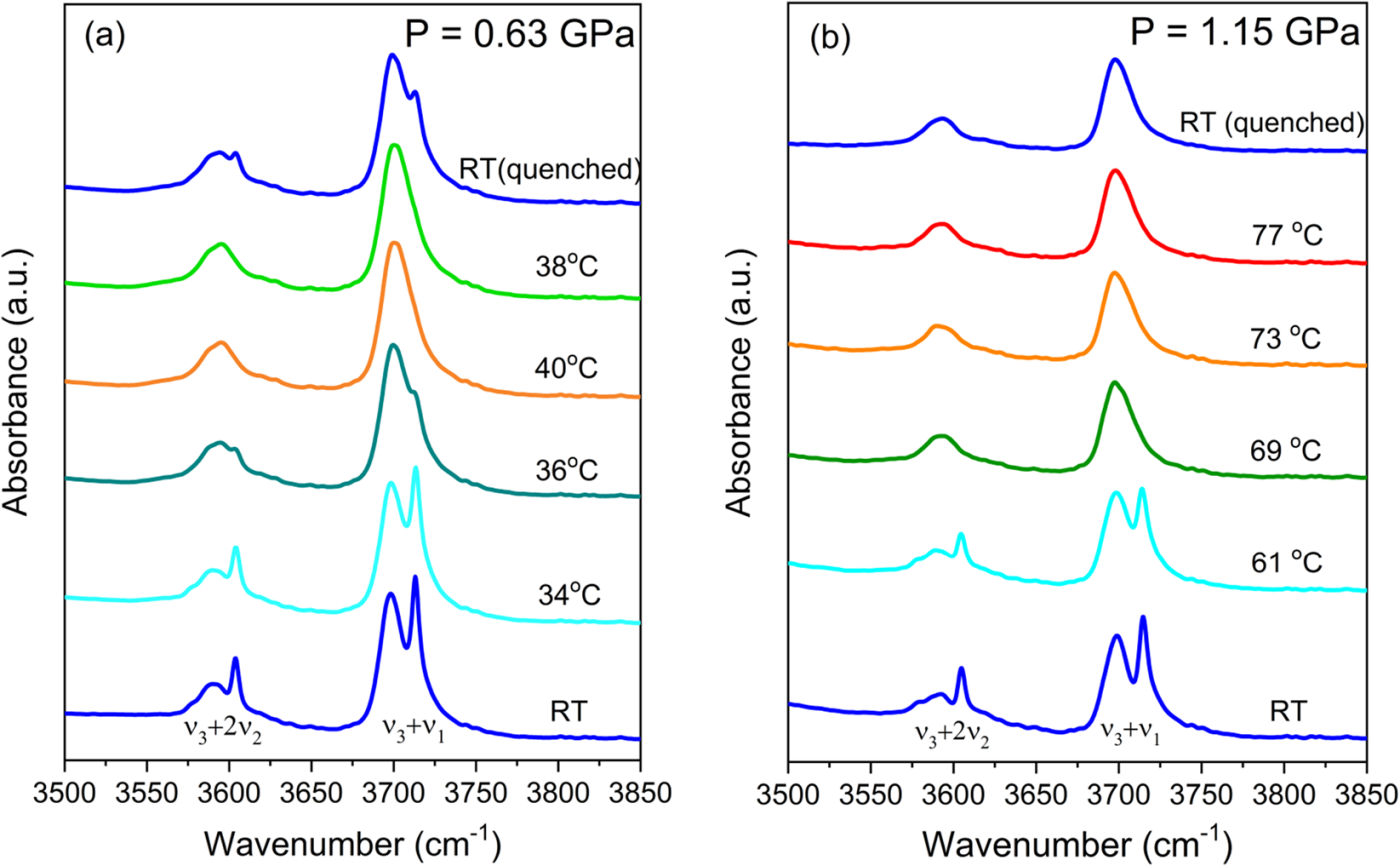
CO_2_ adsorption capacity of ZIF-8 in percentage as a function of pressure and temperature. The data are obtained along a heating sequence at a fixed pressure followed by cooling (quenched) and analyzed using the characteristic combination IR mode of CO_2_. A maximum enhancement (by 80%) of CO_2_ adsorption by heating was achieved at 1.15 GPa (see text).

The selected variable temperature IR spectra of the CO_2_ combination modes of the CO_2_/ZIF-8 collected at a fixed pressure of 0.63 GPa are shown in Fig. 2a. At room temperature, each combination mode exhibits two components, indicating that the CO_2_ molecules in the sample chamber experience two different environments. According to the CO_2_ phase diagram^33^, the CO_2_ residing outside ZIF-8 should exist as a crystalline solid. As mentioned above, the high-frequency sharp component of each combination mode originates from the solid CO_2_ outside the ZIF-8 framework. The CO_2_ molecules adsorbed inside the framework of ZIF-8, on the other hand, give rise to the broader low-frequency component. At 36 °C, both the high-frequency components of (ν_3_ + 2ν_2_) and (ν_3_ + ν_1_) modes become less intense, while the low-frequency component of each mode becomes broader, suggesting that the solid CO_2_ residing outside the framework undergoes a solid-to-fluid transition at current temperature and pressure. Markedly reduced intensity of the peak associated with solid CO_2_ implies that a significant portion of the CO_2_ that initially resides outside the framework at room temperature has now entered the framework at this temperature. Upon further heating at 40 °C, the intensity of the high-frequency component representing solid CO_2_ vanishes completely, indicating that all the solid CO_2_ initially residing outside the ZIF-8 framework has largely entered the framework due to the temperature enhanced fluidity. After cooling the DAC system down to room temperature, the higher frequency component of the doublet of both (ν_3_ + 2ν_2_) and (ν_3_ + ν_1_) modes reappears, but the intensity is much weaker compared to its low-frequency counterpart, suggesting that the CO_2_ outside ZIF-8 recrystallizes upon cooling. This observation unambiguously indicates that after the heating cycle, the number of CO_2_ molecules adsorbed inside ZIF-8 at room temperature is larger than that before heating. The ratio of CO_2_ amount adsorbed inside the framework to those outside ZIF-8 can be estimated quasi-quantitatively from the integrated peak areas obtained by spectral deconvolution.

The two overlapping bands of the (ν_3_ + ν_1_) mode in each spectrum of the CO_2_/ZIF-8 at different temperatures and under a fixed pressure of 0.63 GPa were deconvoluted (Supplementary Fig. S2) and the normalized peak areas are listed in Supplementary Table S1. Before heating, the fraction of CO_2_ inside the ZIF-8 at room temperature is 55%; it then increases to 62% upon heating to 36 °C. At 40 °C, the normalized peak area representing CO_2_ inside the framework reaches 78%, and the rest of the CO_2_ remains as a fluid outside the framework (Supplementary Fig. S2). Upon cooling down to room temperature, the normalized peak area of the adsorbed CO_2_ decreases to 62%. Thus, the amount of CO_2_ adsorbed in the framework at room temperature and a pressure of 0.63 GPa increased by 13% compared to that before heating. It is thus apparent that simultaneously applying high temperature and pressure enhances ZIF-8’s CO_2_ adsorption capability. More importantly, a larger amount of CO_2_ is trapped irreversibly inside the framework upon cooling to room temperature. Such permanent heating-enhanced CO_2_ adsorption of ZIF-8 framework under a confined condition has not been observed for any other MOF materials to the best of our knowledge.

**Figure 3:**
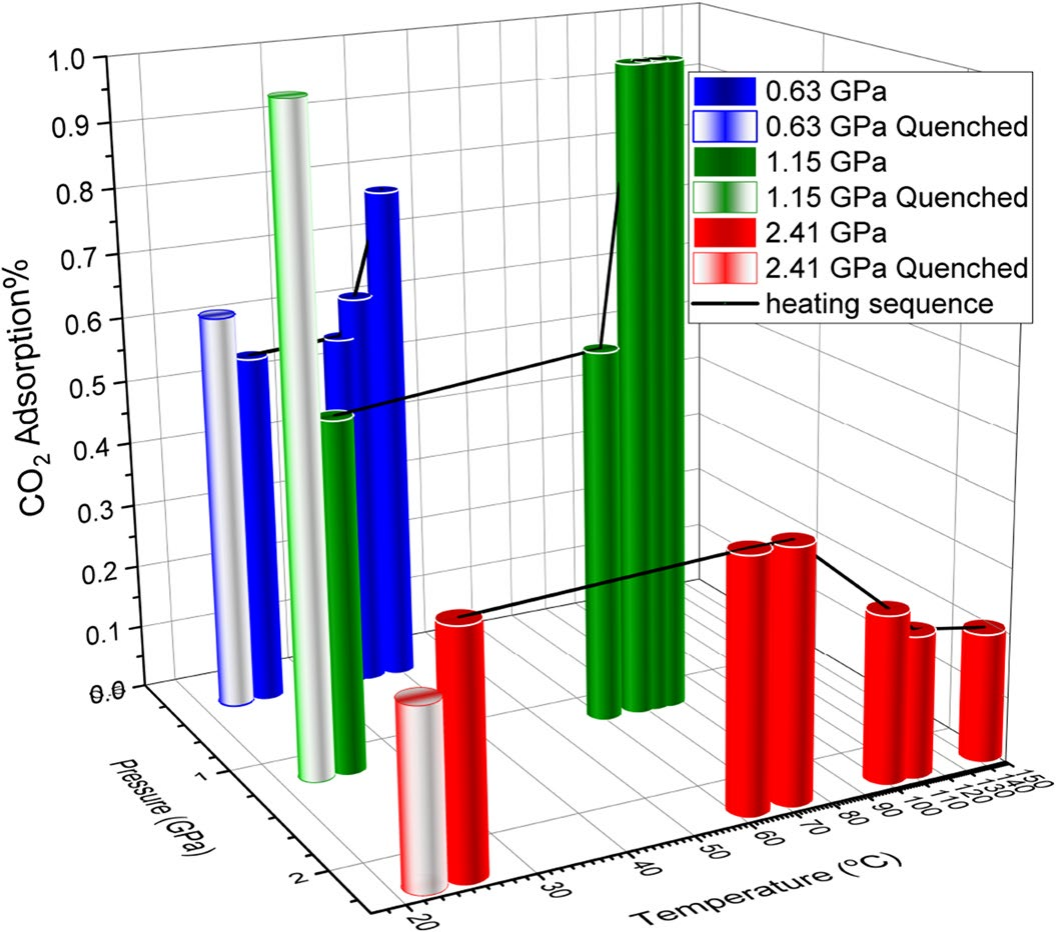
CO_2_ adsorption capacity of ZIF-8 in percentage as a function of pressure and temperature. The data are obtained along a heating sequence at a fixed pressure followed by cooling (quenched) and analyzed using the characteristic combination IR mode of CO_2_. A maximum enhancement (by 80%) of CO_2_ adsorption by heating was achieved at 1.15 GPa (see text).

To further explore and optimize the synergetic effect of high pressure and high temperature on enhancing CO_2_ uptake, additional variable temperature experiments at different pressures were also performed. The selected variable temperature IR spectra of the CO_2_/ZIF-8 at a fixed pressure of 1.15 GPa are illustrated Fig. 2b. At 61 °C, the IR spectrum with sharp (ν_3_ + 2ν_2_) and (ν_3_ + ν_1_) modes clearly shows that CO_2_ inside the sample chamber remains solid at this pressure. When heated to higher temperatures such as 69, 73 and 77 °C, all solid CO_2_ appears melted with most of the CO_2_ molecules transported into the framework of ZIF-8 by diffusion. Upon cooling from 77 °C down to room temperature, both (ν_3_ + 2ν_2_) and (ν_3_ + ν_1_) modes remain as a single broad peak, suggesting that no solid CO_2_ phase is recovered. This result is significant as at room temperature and 1.15 GPa, any CO_2_ existing outside ZIF-8 framework should be in solid phase. Among other additional CO_2_ adsorption measurements following different P–T paths, we found that 1.15 GPa is close to an optimized pressure at which CO_2_ adsorption enhancement is maximized upon heating to a temperature near the melting point of CO_2_. Such a mild pressure–temperature condition can be readily realized in other devices such as multi-anvil press to allow scaling up. The normalized peak area (Supplementary Table S2) indicates that at 1.15 GPa and room temperature, 54% of the total CO_2_ are initially adsorbed inside the cages of ZIF-8 and 46% of CO_2_ resides outside the framework as a solid. Apparently upon heating followed by cooling back to room temperature, all the CO_2_ molecules have now migrated and been trapped inside the ZIF-8 framework. A careful inspection reveals that upon cooling down to room temperature, the peak due to the (ν_3_ + 2ν_2_) mode has an asymmetric peak profile; the deconvoluted spectrum (Supplementary Fig. S3) shows a small broad peak with a Gaussian profile at the high-frequency side of the main peak, indicating that there are two non-equivalent CO_2_ inside ZIF-8 (see discussion below). The heat treatment at 1.15 GPa leads to an 85% increase in the amount of adsorbed CO_2_ in ZIF-8, compared to only 13% enhancement of CO_2_ uptake under 0.63 GPa. We further explored the CO_2_ uptake behaviour in ZIF-8 framework under other P–T conditions extensively (up to 2.4 GPa and over 145°C) and found 1.15 GPa is the closest to an optimal pressure under which the heating-enhanced CO_2_ adsorption achieves the maximum. These findings are summarized in Fig. 3.

## Discussion

**Figure 4:**
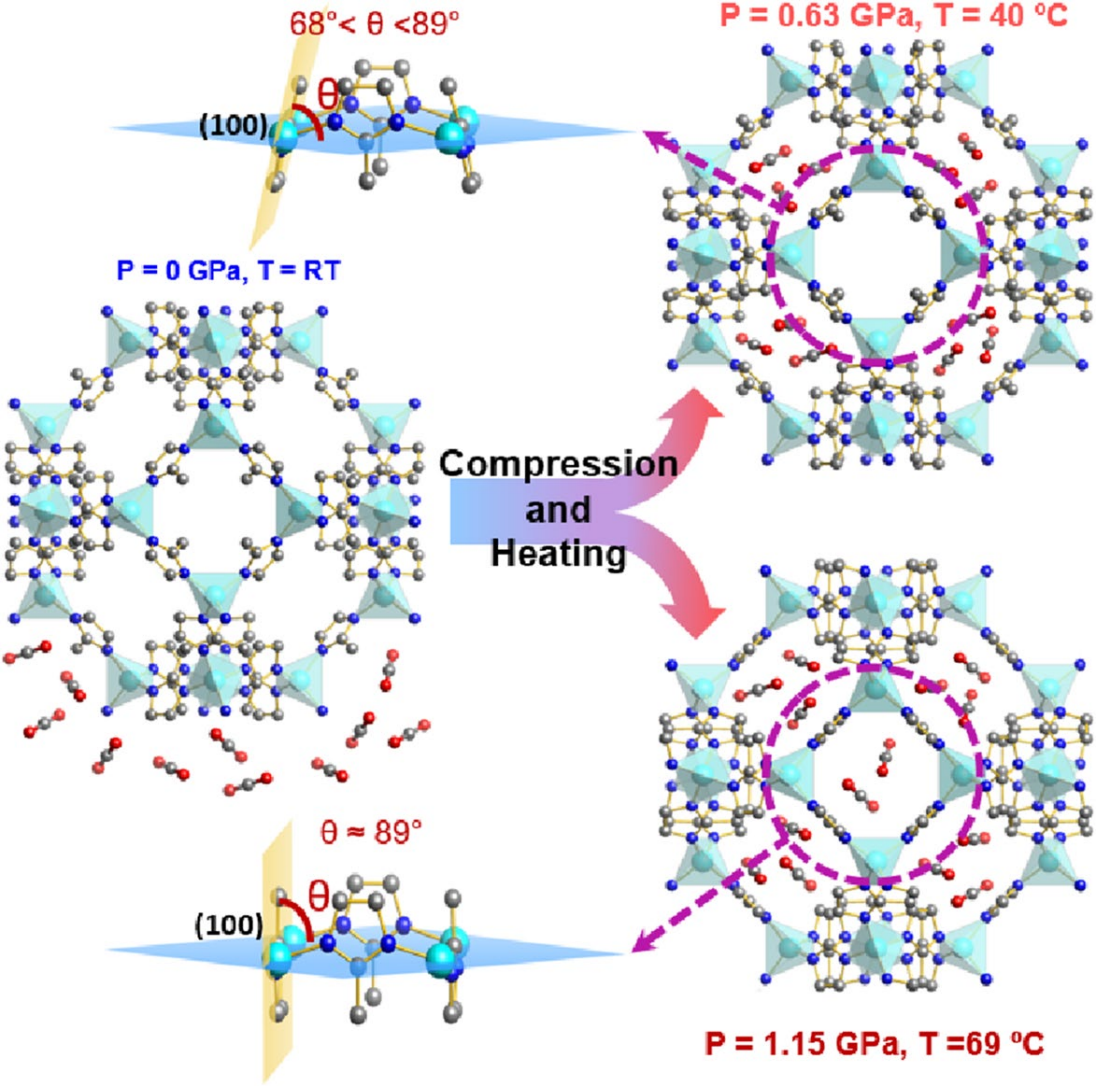
Schematics of CO_2_ adsorption and structural change in the CO_2_ loaded ZIF-8 framework from near ambient pressure and room temperature (left) to 0.63 GPa and 40 °C (upper right) and to 1.15 GPa and 69 °C (lower right). The pressure-dependent “gate” opening effect is illustrated by the rotation angle of the methylimidazole rings (see text).

The substantial difference in adsorptive capacity improvement under different pressures is intriguing and can be closely attributed to the framework structure under different pressures and temperatures. Previous work by Moggach et al.^21^ showed that high external pressure can increase the effective pore size of ZIF-8 by changing the linker orientation, which is so called “gate opening” effect. Hobday et al. further established a correlation between the pressure and the rotation angle (θ) of the imidazole rings describing the pore opening (Fig. 4)^26^. Such rotation of the linker results in an increase in the unit cell volume and solvent-accessible volume^6,26^. In the present case, it appears that at 0.63 GPa, the pressure is not high enough to fully rotate the imidazole ring to open the gate completely. Consequently, at this pressure, the CO_2_ uptake has not reached the maximum even though the fluidity of CO_2_ is significantly enhanced by heating. The work by Moggach et al.^21^ elegantly demonstrated that ZIF-8 transforms to a new phase at 1.47 GPa (a pressure close to 1.15 GPa explored in this study) where both the pore volume and the size of the linking channels increase. At 69 °C and 1.15 GPa, we believe that the small and broad high-frequency peak in the deconvoluted spectrum (Supplementary Fig. S3) represents 22% of CO_2_ molecules that are adsorbed in the channels surrounding each sodalite cage and are distinctively different than those 78% in the cages. These channels are otherwise blocked by imidazole linkers and not accessible at the ambient pressure. The CO_2_ molecules adsorbed in the channels are tightly confined compared to those inside the cages. Consequently, the CO_2_ vibrations appeared at higher frequencies due to the strong confinement. The fact that no solid phase of CO_2_ reforms upon cooling down to room temperature implies that all the CO_2_ molecules are trapped tightly inside ZIF-8 due to the strong interaction of CO_2_ with the framework under pressure. The combined pressure–temperature effect on the structural details of ZIF-8 framework is illustrated in Fig. 4.

To further confirm that fluidity of CO_2_ under applied pressure by heating is critically important to the large enhancement of CO_2_ uptake of ZIF-8, we heated the CO_2_/ZIF-8 mixture in a DAC at temperature up to above 145 °C under a higher pressure of 2.41 GPa. CO_2_ phase diagram shows that the free CO_2_ residing outside the framework should remain as a solid in the entire T, P range employed. Indeed, the high-frequency component of the (ν_3_ + ν_1_) mode representing solid CO_2_ persists in the spectra throughout the entire temperature range (Supplementary Fig. S4). No CO_2_ uptake enhancement was observed either during heating or upon cooling down to room temperature (Supplementary Fig. S4). These results unambiguously indicate that neither heating solid CO_2_ nor applying pressure alone can sufficiently improve the CO_2_ adsorptive capability of ZIF-8. Careful inspection of the room temperature spectra acquired at 2.41 GPa before and after heating reveals that the intensity of the high-frequency peak corresponding to solid CO_2_ actually becomes slightly stronger with respect to the low-frequency component due to the CO_2_ inside ZIF-8, implying that the CO_2_ adsorptive ability is slightly reduced after the heat treatment (Supplementary Fig. S4). A possible reason is that the framework might have been partially damaged by exposing the ZIF-8 to harsher conditions (i.e. 2.41 GPa and 145 °C), resulting in a slightly reduced porosity for adsorption. The quantitative CO_2_ intake enhancement and detailed CO_2_ adsorption mechanism at simultaneously high P–T conditions should be verified by synchrotron single crystal diffraction measurements as well as computational modelling in the future. Moreover, whether the synergetic P–T effect is applicable to other MOFs in different classes invites more systematic study on other MOFs.

## Conclusions

In summary, using in situ IR spectroscopy, we demonstrate that simultaneously applying moderate temperature and pressure to a CO_2_/ZIF-8 mixture can remarkably enhance the CO_2_ adsorptive capacity of ZIF-8. Specifically, the thermal treatment was applied simultaneously at high pressures up to 2.41 GPa. The optimized T, P condition established in this study is 69 °C and 1.15 GPa, under which an 85% increase in CO_2_ uptake is achieved. Such a drastic enhancement of CO_2_ adsorption ability is attributed to the synergetic effect of high T and P. High temperature significantly enhances the transport property of CO_2_, allowing CO_2_ to effectively diffuse into the framework of ZIF-8. Meanwhile, high mechanical pressure changes the effective pore size and shape as well as the linker orientation to open the “gate”, facilitating the CO_2_ adsorption. The change in the linker orientation also makes the channels connecting the cages accessible to CO_2_ as additional adsorption sites at high pressure. This work demonstrates that gas adsorption capability of MOFs and ZIFs can be drastically improved by applying moderate pressure and temperature, making ZIFs a promising class of materials for practical CO_2_ storage applications.

## Methods

**Figure 5:**
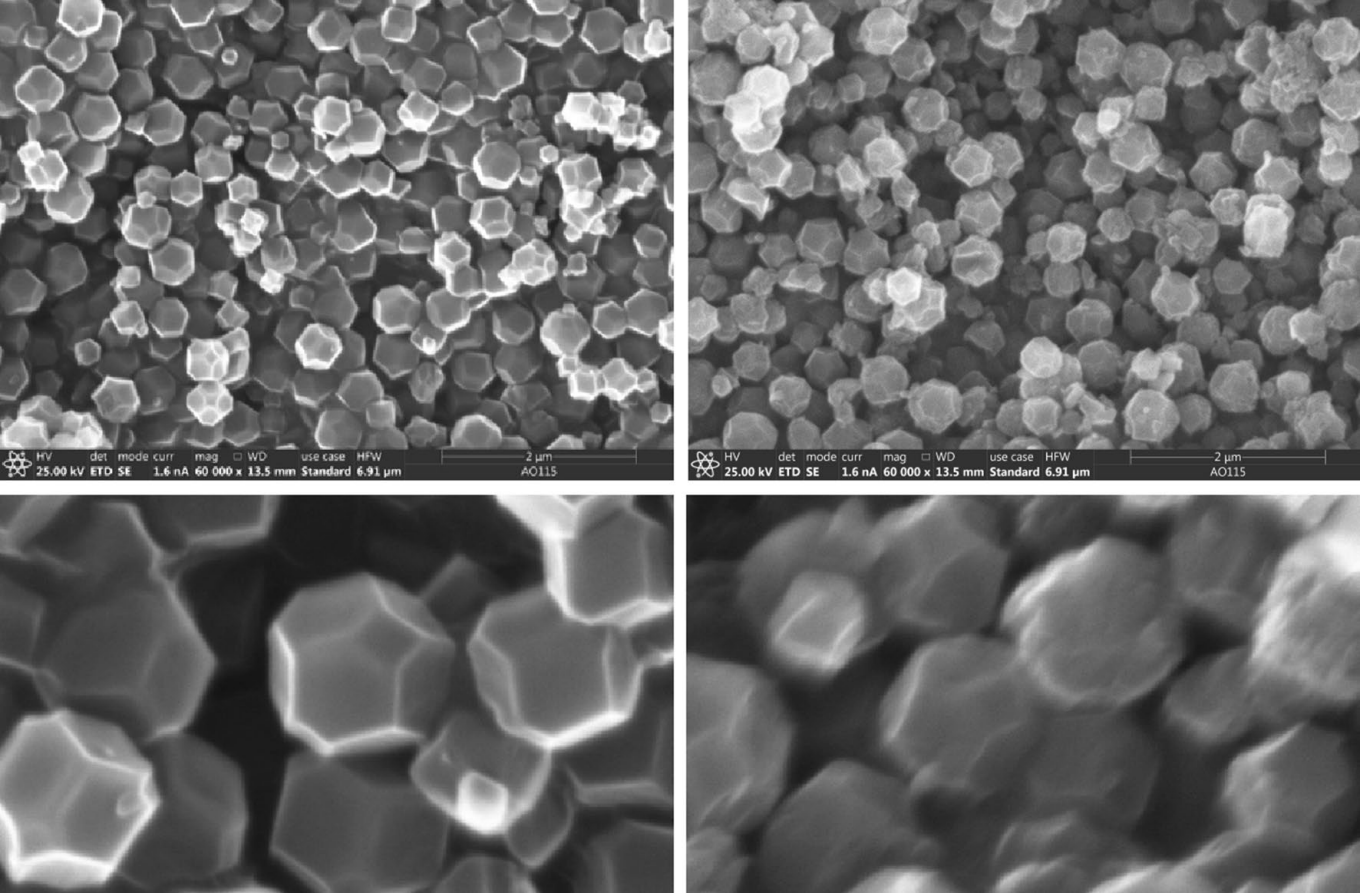
SEM images of as-made (left column) and activated (right column) ZIF-8 samples at different magnifications.

ZIF-8 was synthesized solvothermally and activated by following the literature method^36^. Briefly, A mixture of 2.5 mmol Zn(NO_3_)_2_·6H_2_O (Alfa Aesar, 98%) and 0.15 mol 2-methylimidazole (Sigma-Aldrich, 98%) were dissolved in 100 mL of deionized water and stirred at room temperature for 24 h. The resulting white crystals were harvested via vacuum filtration and washed with 10 mL of methanol for three times. Activation of ZIF-8 samples was achieved by degassing via dynamic vacuum and heating for 8 h at 150 ℃. The identity and purity of the activated product were verified by powder X-ray diffraction (PXRD) to show the successful removal of the solvent to achieve an activated (empty) ZIF-8 framework. (Supplementary Fig. S5). The PXRD pattern was recorded using a Inel CPS X-ray diffractometer operating with Cu Kα radiation (λ = 1.5406 Å). The reflections were collected at 2θ values ranging between 5 and 40°, using an increment of 0.01° with an acquisition rate of 4°/min. Thermogravimetric Analysis (TGA) measurements were performed by Beijing HENVEN HQT-3 instrument. The sample was heated from 25° to 800 °C at a rate of 10 °C/min under a N_2_ atmosphere. The TGA profile of the activated sample (Supplementary Fig. S6) shows no weight loss below around 600 °C, which clearly indicates that all the solvent molecules were removed during activation process and the activation is complete. The weight loss at the temperature above 600 °C is due to the decomposition^36^, indicating the ZIF-8 is stable up to c.a. 600 °C. Scanning Electron Microscopy (SEM) measurements were conducted using a Thermo Scientific Apreo S SEM instrument operating at 30 kV. SEM images of as-made and activated ZIF-8 samples are shown in Fig. 5. ZIF-8 crystals display a consistent truncated octahedral shape with uniform particle dimensions, in agreement with previously reported studies^36^. The surface area of activated ZIF-8 was measured using BET method with a BELSORP-MAX instrument with N_2_ (99.999%) at 77 K. Prior to N_2_ isotherm measurement, 100 mg of ZIF-8 was achieved by degassing via dynamic vacuum and heating for 8 h at 150 ℃. N_2_ adsorption experiments at pressures up to 1 bar was then performed on activated ZIF-8 by the BELSORP-MAX. The N_2_ adsorption isotherms measured for activated ZIF-8 sample is shown in Supplementary Fig. S7. The BET area derived is 1686.7 m^2^/g, surpassing the best value (i.e., 1550 m^2^/g) reported in a previous literature^36^, indicating the successful activation and excellent porosity of ZIF-8 achieved in this study.

To carry out in situ high T-P experiments, a DAC (Supplementary Fig. S8) equipped with a pair of type II diamonds (600 µm culet size) was used for all IR measurements. The sample chamber was prepared on a stainless-steel gasket, with 60–80 µm in thickness and 300 µm in diameter. A few ruby chips were pre-loaded into the sample chamber for pressure calibration^37^. For sample preparation, in order to maximize the CO_2_ loading, the sample chamber was firstly half filled with activated (empty) ZIF-8. The piston side of the DAC was then immersed in a liquid nitrogen bath to cool down the system. During this process, the sample chamber was covered with a plastic film to avoid moisture condensation. When the temperature of the gasket was below the melting point of dry ice (i.e., < 78.5 °C), the plastic film was removed, and the CO_2_ gas was introduced into the sample chamber. Upon closing the DAC, a low pressure is needed to hold CO_2_ in the sample chamber for further measurements. The FTIR spectrometer used is customized for in situ high pressure measurements, with the details described in previous publications^38^. In this work, to apply high pressure and high temperature simultaneously and take IR measurement in situ, the regular DAC stage for room temperature measurement was replaced by a custom-designed heating stage, which is composed of a resistively heated copper cell holder, and a glass wool board for heat insulation. By placing the DAC onto this stage, high temperature (up to ~ 500 °C) could be achieved by powering up the heating element with AC current from a power supply with a temperature controller (omega iSeries) to regulate the heat delivery and to control the temperature of the DAC. Temperature is measured by attaching a calibrated thermocouple to the back of the diamond. The accuracy of the temperature measurement is ± 2.3 °C in the temperature range from 50–80 °C and ± 5.0 °C from 80 to 200 °C.

### Supplementary Information

Supplementary Information.

## Data Availability

All data generated or analysed during this study are either included in this published article and its supplementary information files or are available from the corresponding author on reasonable request.
