# Supplementary material for: Enhancing CO2 adsorption capacity of ZIF-8 by synergetic effect of high pressure and temperature
Source: Sci Rep. 2023 Oct 16;13:17584. doi: 10.1038/s41598-023-44960-4 (PMC10579389; doi:10.1038/s41598-023-44960-4)
Supplement: Supplementary file 1 — Supplementary Information. [file 41598_2023_44960_MOESM1_ESM.pdf]

SUPPLEMENTARY INFORMATION

Enhancing CO_2 Adsorption Capacity of ZIF-8 by Synergetic Effect of High Pressure and Temperature

*Shan Jiang[a], Jingyan Liu[a], Jiwen Guan[b], Xin Du[c], Shoushun Chen[c], Yang Song[*a, b] and Yining Huang[*a]*

[a] Department of Chemistry, The University of Western Ontario, London, ON N6A 5B7, Canada

[b] Department of Physics and Astronomy, The University of Western Ontario, London, ON N6A 5B7, Canada

[c] Lanzhou Magnetic Resonance Center, College of Chemistry and Chemical Engineering, Lanzhou University, Lanzhou 730000, China

***Corresponding Authors:** Yang Song, Email: yang.song@uwo.ca; Yining Huang, Email: yhuang@uwo.ca

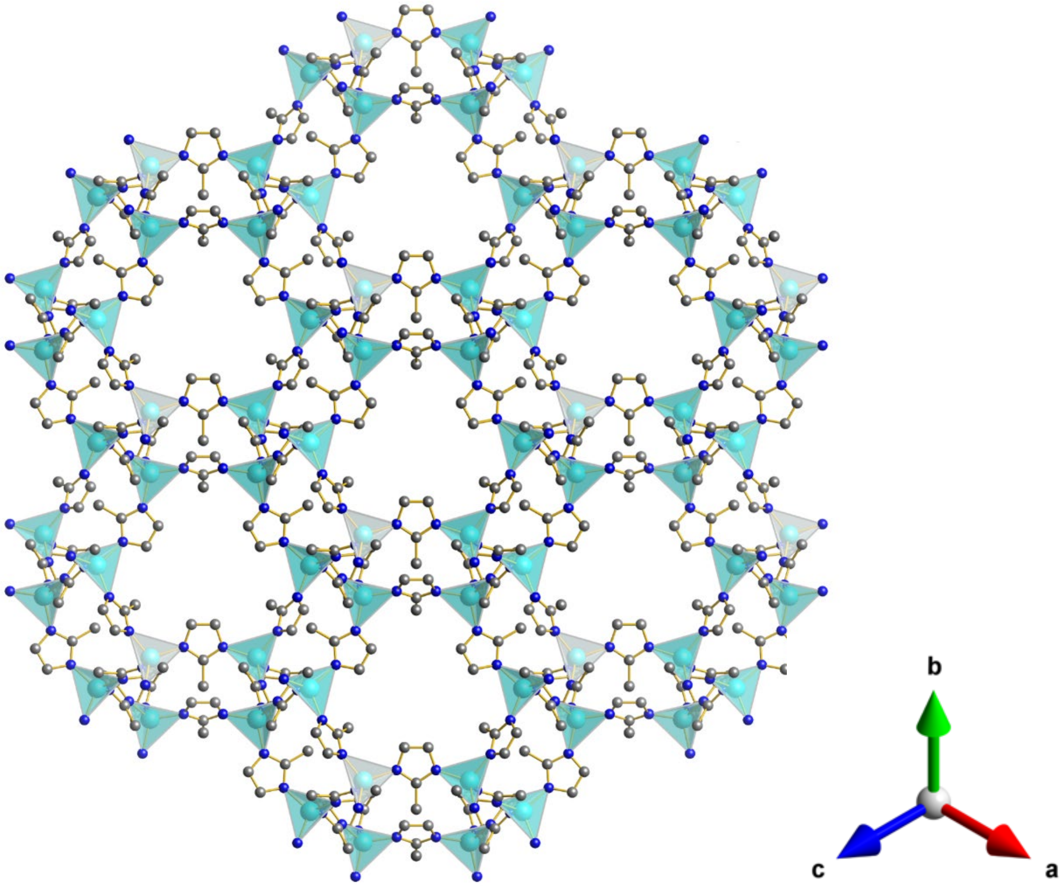

Supplementary Figure S1. Framework topology of ZIF-8.

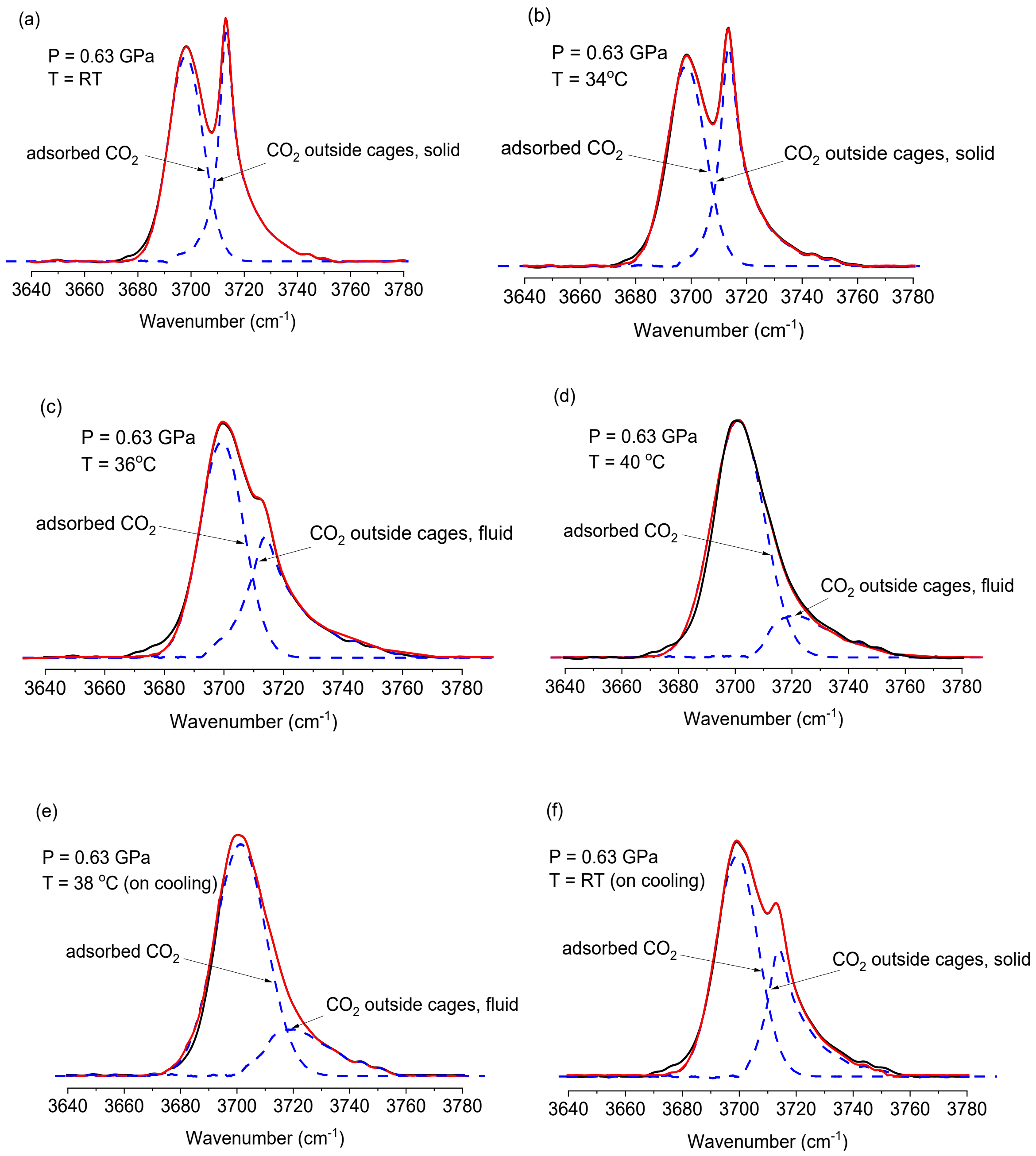

Supplementary Figure S2. (a)-(f) Deconvolution of CO_2 ν_3+ν_1 mode on the FTIR spectra of CO_2 loaded ZIF-8 at 0.63 GPa and various temperatures. In all figures, the black solid line represents the actual spectrum, the red solid line is the fitted spectrum. The blue dash lines represent different species of CO_2.

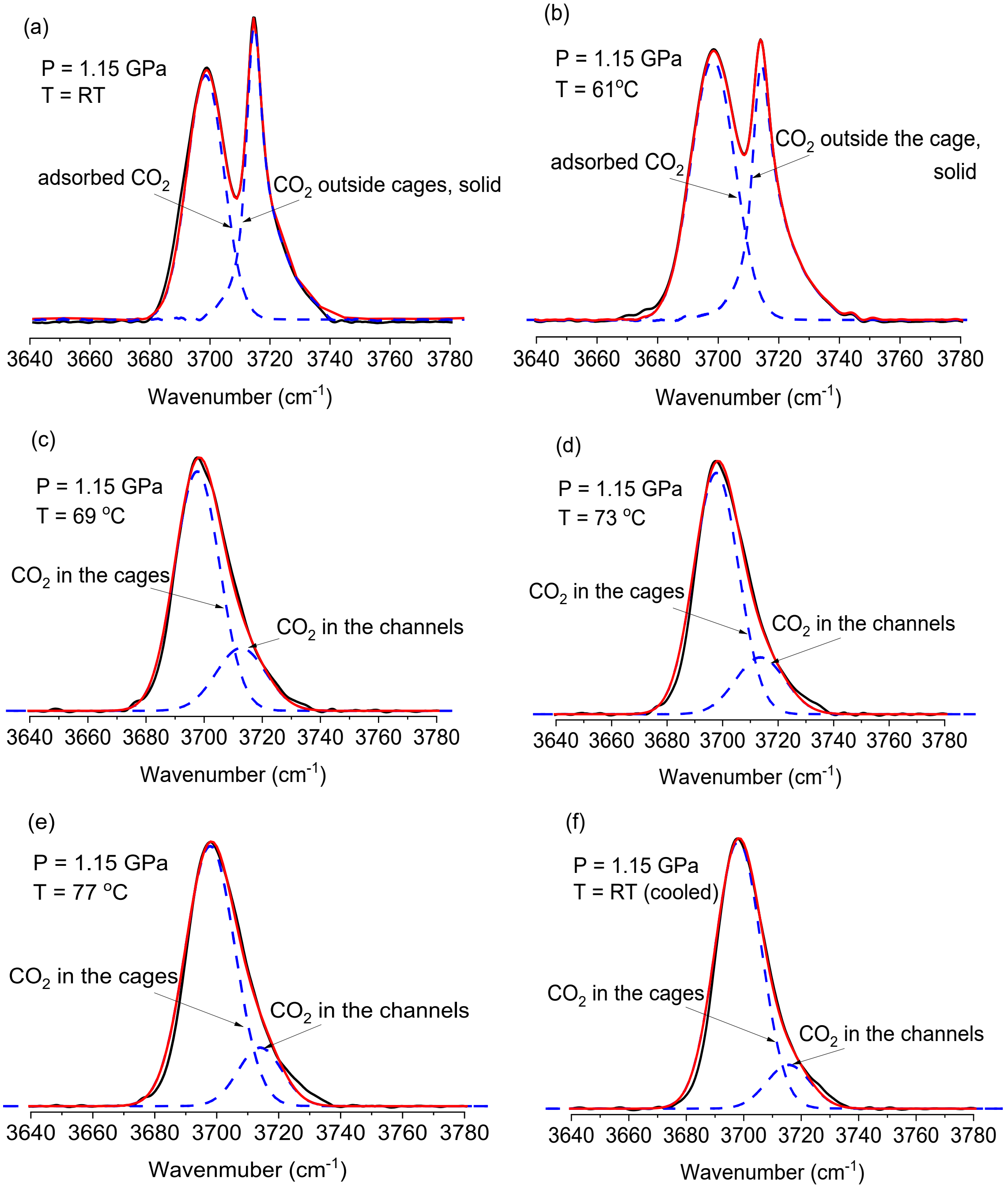

Supplementary Figure S3. (a)-(f) Deconvolution of CO_2 ν_3+ν_1 mode on the FTIR spectra of CO_2 loaded ZIF-8 at 1.15 GPa and various temperatures. In all figures, the black solid line represents the actual spectrum, the red solid line is the fitted spectrum. The blue dash lines represent different species of CO_2. In figure (c)-(f), the main peak at lower frequency could be assigned as CO_2 molecules adsorbed in the central cage, whereas the higher frequency peak is thought to be attributed to the CO_2 adsorbed in the channels surrounding the central cage.

Supplementary Table S1. Normalized peak area of CO_2 $\nu_3 + \nu_1$ mode at 0.63 GPa and various temperatures in CO_2 loaded ZIF-8.

Temperature	RT	34 °C	36 °C	40 °C	38 °C	RT (quenched)
Peak area of adsorbed CO_2 (%)	55	56	62	78	79	62
Peak area of CO_2 outside framework (%)	45	44	38	22	21	38

Supplementary Table S2. Normalized peak area of CO_2 $\nu_3 + \nu_1$ mode at 1.15 GPa and various temperatures in CO_2 loaded ZIF-8.

<table>
<tr><th colspan="2">Temperature</th><th>RT</th><th>61 °C</th><th>69 °C</th><th>73 °C</th><th>77 °C</th><th>RT (quenched)</th></tr>
<tr><td rowspan="2">Peak area of adsorbed CO_2 (%)</td><td>in the central cages</td><td>54</td><td>58</td><td>78</td><td>79</td><td>83</td><td>83</td></tr>
<tr><td>in the channels</td><td>-</td><td>-</td><td>22</td><td>21</td><td>17</td><td>17</td></tr>
<tr><td colspan="2">Peak area of CO_2 outside framework (%)</td><td>46</td><td>42</td><td>-</td><td>-</td><td>-</td><td>-</td></tr>
</table>

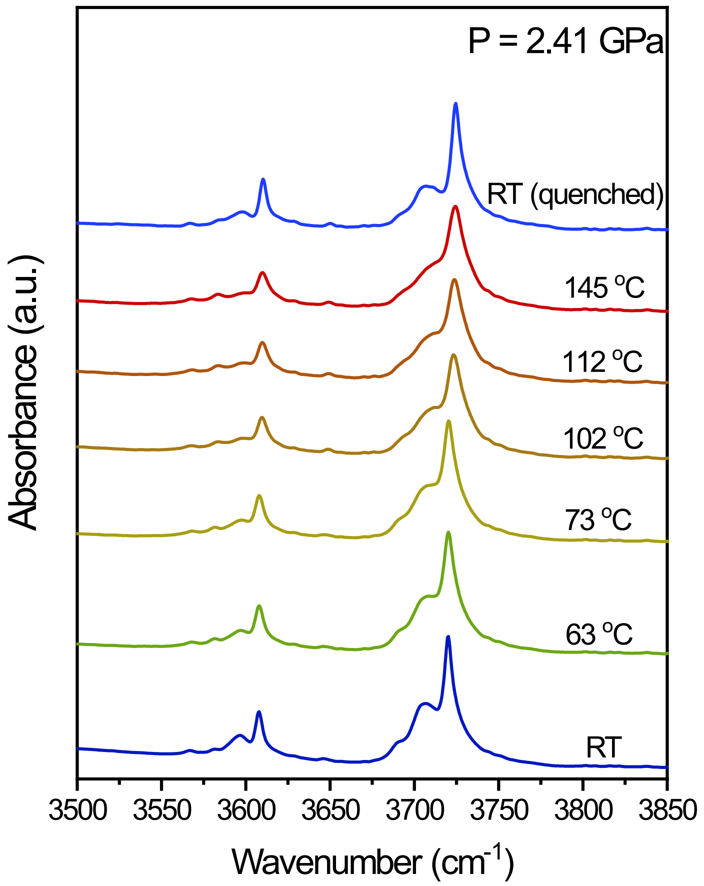

Supplementary Figure S4. Variable temperature IR spectra of CO_2 combination modes of the CO_2/ZIF-8 sample at 2.41 GPa.

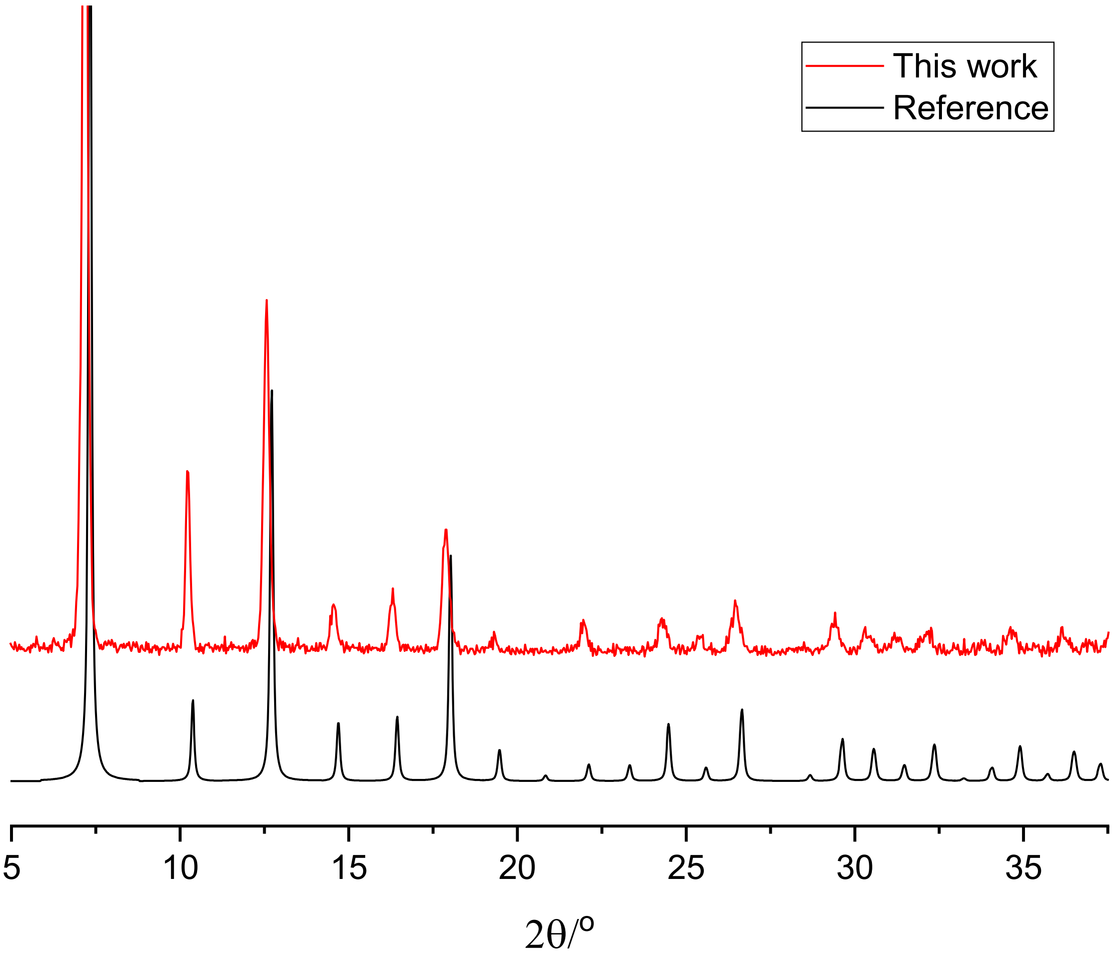

Supplementary Figure S5. PXRD pattern of activated ZIF-8 (red trace) at ambient conditions in comparison with simulated using reference (black trace).

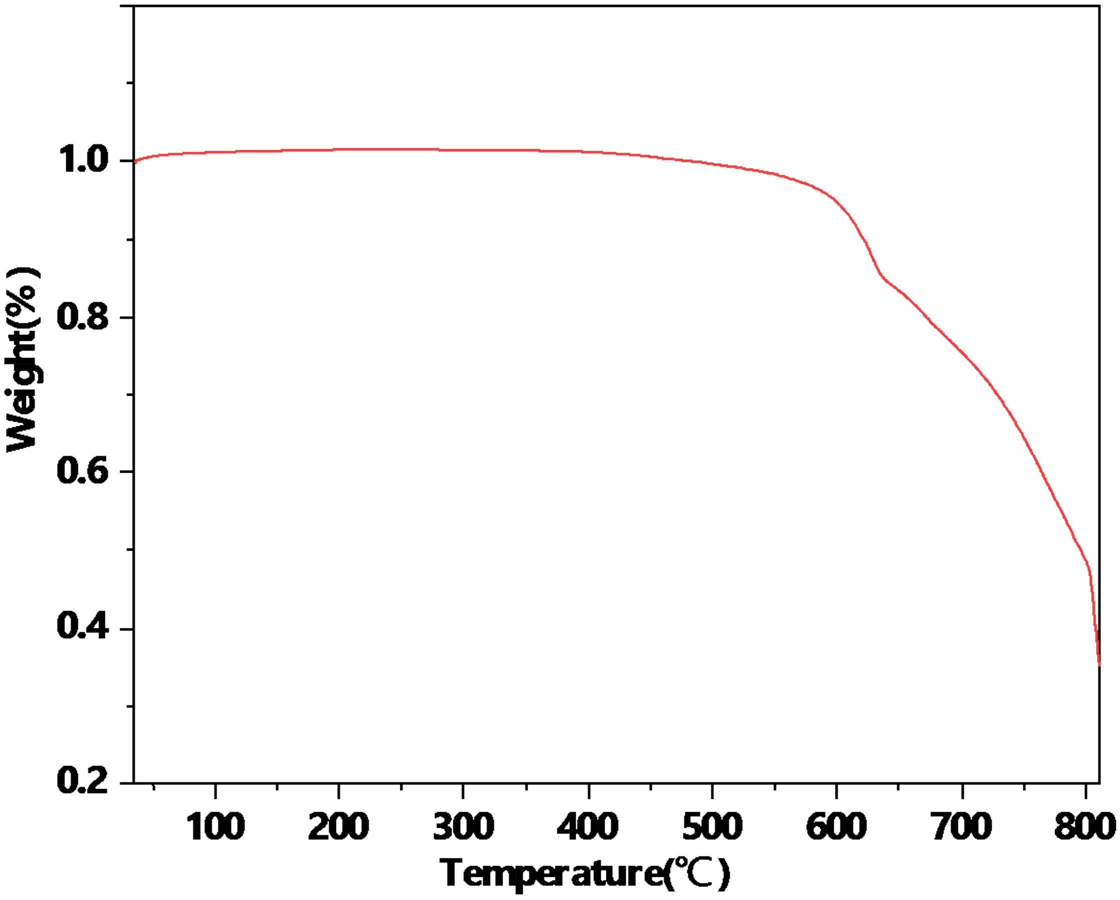

Supplementary Figure S6. TGA profile for the activated ZIF-8. The TGA profile of the activated sample shows no weight loss below around 600 °C, clearly indicating that the activation is complete.

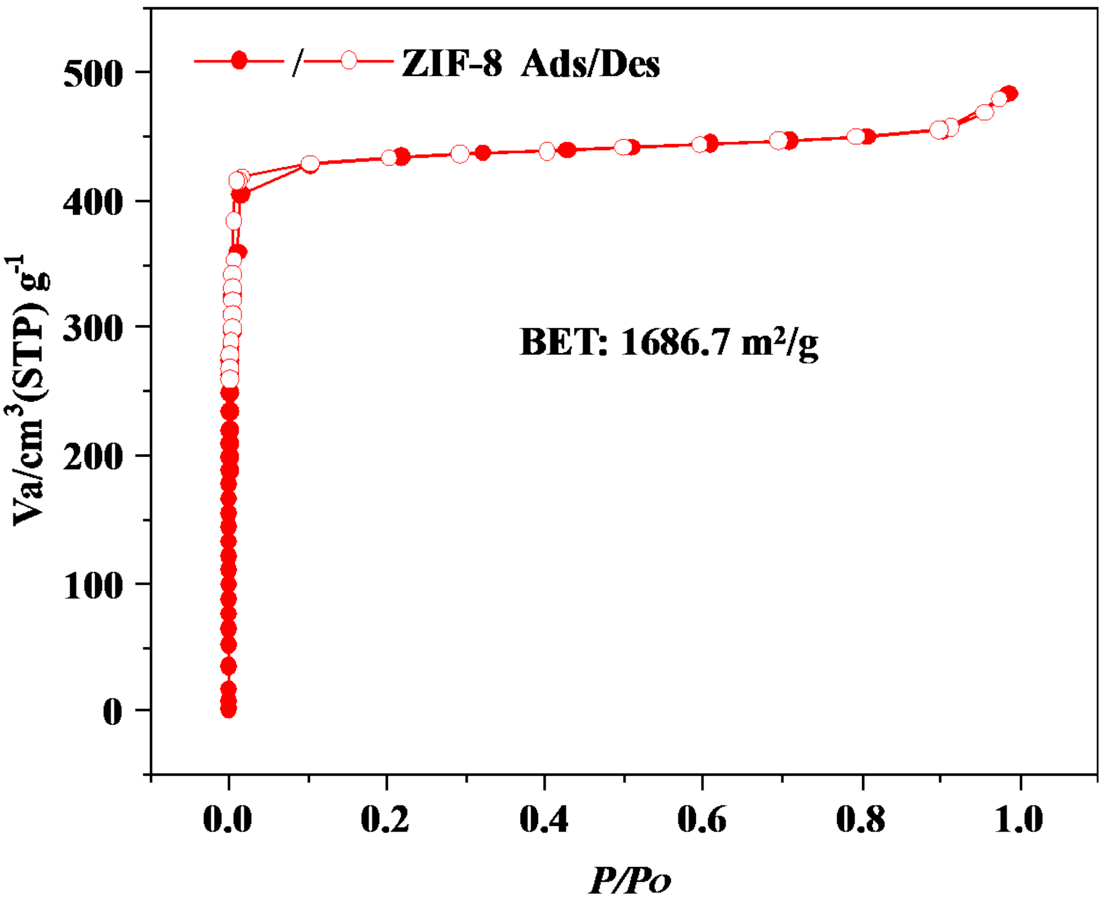

Supplementary Figure S7. The N_2 adsorption isotherms measured for activated ZIF-8 sample.

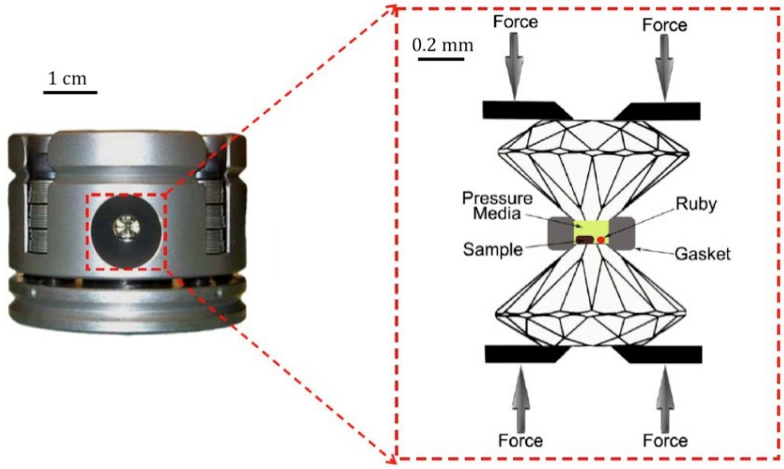

Supplementary Figure S8. Photo and schematics of a symmetric diamond anvil cell. Reproduced from “ Y. Song and Z. Dong, Novel Pressure-Induced Structural Transformations of Inorganic Nanowires in “Nanowires”, ed. A. Hashim, Intech (ISBN 978-953-307-327-9), pp527-552, 2011.” with permission.
